# Effects of new urban motorway infrastructure on road traffic accidents in the local area: a retrospective longitudinal study in Scotland

**DOI:** 10.1136/jech-2016-207378

**Published:** 2016-06-08

**Authors:** Jonathan R Olsen, Richard Mitchell, Daniel F Mackay, David K Humphreys, David Ogilvie

**Affiliations:** 1 Centre for Research on Environment, Society and Health, Institute of Health and Wellbeing, University of Glasgow, Glasgow, UK; 2 Institute of Health and Wellbeing, College of Medical, Veterinary and Life Sciences, University of Glasgow, Glasgow, UK; 3 Department of Social Policy and Intervention, University of Oxford, Oxford, Oxfordshire, UK; 4 MRC Epidemiology Unit, UKCRC Centre for Diet and Activity Research (CEDAR), School of Clinical Medicine, University of Cambridge, Cambridge, Cambridgeshire, UK

**Keywords:** Environmental epidemiology, ACCIDENTS, Outcome Research Evaluation, INJURY, TRAFFIC

## Abstract

**Background:**

The M74 motorway extension, Glasgow, opened in June 2011. One justification for construction was an expectation that it would reduce road traffic accidents (RTAs) on local non-motorway roads. This study evaluated the impact of the extension on the number of RTAs, stratifying by accident severity.

**Methods:**

Data for the period 1997–2014 were extracted from a UK database of reported RTAs involving a personal injury. RTA severity was defined by the level of injury: minor, severe or fatal. RTAs were assigned to (1) the local area surrounding the motorway extension, (2) a comparator area surrounding an existing motorway or (3) a control area elsewhere in the conurbation. Interrupted time-series regression with autoregressive integrated moving average (ARIMA) errors was used to determine longitudinal between-area differences in change in the number of RTAs, which might indicate an intervention effect.

**Results:**

Glasgow and surrounding local authorities saw a 50.6% reduction in annual RTAs (n: 5901 to 2914) between 1997 and 2014. In the intervention area, the number of recorded RTAs decreased by 50.7% (n: 758 to 374), and that of fatal/severe RTAs by 57.4% (n: 129 to 55), with similar reductions in the comparator/control areas. The interrupted time-series analysis showed no significant between-area differences in temporal trends. The reduction of pedestrian casualties was attenuated in the intervention area relative to Glasgow and surrounding authorities.

**Conclusions:**

Reduction in RTAs was not associated with the motorway extension. Our findings suggest that in planning future investment, it should not be taken for granted that new road infrastructure alone will reduce RTAs in local areas. Urbanisation is proceeding rapidly worldwide, and evidence of infrastructure changes is lacking; this novel study provides important findings for future developments.

## Introduction

Road traffic accidents (RTAs) are a major public health concern; during 2014 alone, there were 1775 reported deaths on UK roads^1^ and 1.3 million worldwide (2010).^2^ More deprived areas have a higher incidence of traffic-related casualties.^3^ RTAs result from a combination of many factors, including the design of the road network, other environmental factors such as urbanicity,^4^ weather, vehicles, road users^5^ and how these all interact.^6^ The number and proportion of accidents are greatest on roads where traffic speeds are limited to 40 mph or less (72% of total casualties), whereas motorways—which in the UK contain 21% of all traffic—account for 5.4% of fatalities and 2.7% of casualties.^1^ These statistics suggest that motorways provide a safer infrastructure for traffic flow.

Overall, numbers of road accidents in the UK are decreasing^7^ due to factors such as improved road safety and management. This is despite increases in vehicle numbers and journeys.^3^ Nevertheless, reducing the number of RTA casualties is a key priority for governments in the UK and around the world.^8^ Interventions aimed at RTA reduction can be broadly characterised as educational, legal or engineering/infrastructural interventions. Many such strategies do produce falls in the number of accidents.^9^ However, there is comparatively little evidence indicating whether construction of new transport infrastructure reduces the number of RTAs. The best available evidence relates to now rather dated evidence, from Norway (1990s), Sweden (1980s), Denmark (1990s), UK (1960s) and USA (1970s and 1990s) which found a before and after decrease in RTAs of 7% (95% CI −7% to −9%).^10^

Our study was a natural experiment, exploiting the construction of 5 miles of new motorway running through Glasgow, Scotland, which opened in June 2011. A new road was constructed mainly above existing roads and dwellings, not replacing an existing arterial road. There were changes to existing road layouts which led to new motorway junctions. The so-called M74 extension was built to relieve congestion on existing motorways in the city and was controversial: an independent report recommended against the proposal, advising that it would be likely to have very serious undesirable results for local communities.^11^ But the government at the time maintained that it would be beneficial by improving road safety and thus reducing RTAs in the local area.^12^ This provided a natural experimental opportunity to explore the arguments for and against the investment in new urban road infrastructure—arguments which, at the time, lacked a clear evidence base—and to contribute new evidence relevant to similar future proposals, particularly in countries going through the ‘motorisation transition’ in which new highways are more frequently constructed. The specific aims of this study were to evaluate the impact of the M74 motorway extension in terms of (1) changes in the number of RTAs during construction and following its opening, (2) differences in these outcomes by accident severity and (3) changes in the distribution of casualties between types of road user (pedestrian, driver, passenger or cyclist).

## Methods

### Data source

STATS19 data were obtained for the period 1997–2014 from the UK Department for Transport. STATS19 provides routinely collected data about all RTAs in the UK which have resulted in a casualty and have been reported to the police.^13^ Detailed data are provided about each accident including date, casualty severity and location (precise coordinates). Each accident can be linked to a more detailed dataset describing the type of road user (pedestrian, driver, passenger or cyclist) and more information on the accident and the casualty. Casualty severity is preclassified using the following definitions: slight, an accident in which at least one person is slightly injured but no-one is killed or seriously injured; serious, in which at least one person is seriously injured but no-one is killed; and fatal, in which at least one person is killed.^14^

### Design and study areas

**Figure 1:**
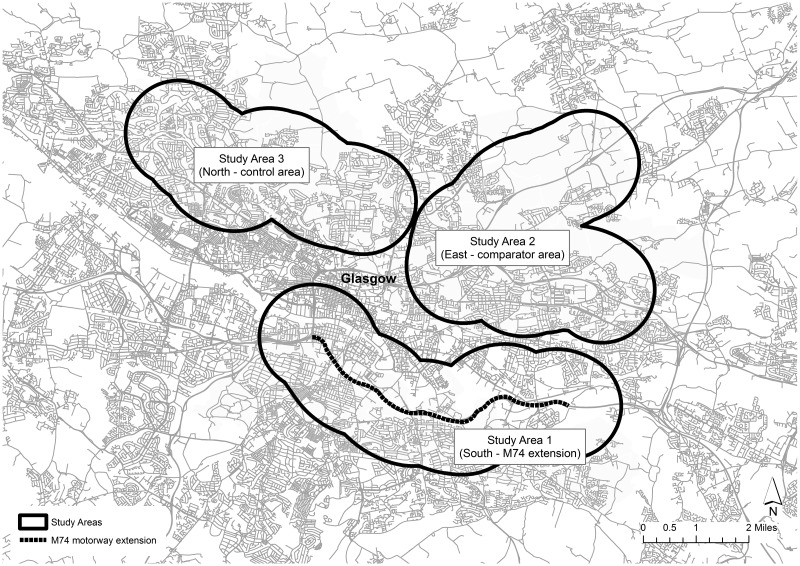
M74 study areas, Glasgow, UK.

This study is a component of a larger evaluation which includes assessment of impacts on active travel, community perceptions and social interaction.^15^ The overall study design entailed three study areas: the local area surrounding the new motorway in the South of the city (intervention area), an area surrounding an existing motorway (the M8) in the East of the city (comparator area) and an area without a motorway, surrounding a suburban railway line in the North of the city (control area). The three areas will be referred to as North, East and South hereafter.^15^ Each area was delineated to be broadly comparable in social, economic and demographic terms, using a 1000 m (0.6 mile) buffer around each of these linear transport structures. A wide buffer was chosen due to the M74 extension being a major new road in the South of the city which linked to other major roads in the West of Scotland and North England. We anticipated that people will travel substantial distances using the vast network of roads to gain access. RTAs were assigned to study areas based on the coordinates of each accident, and changes over time in the number of RTAs were compared between the three areas (figure 1).

For this study, we also included a much larger reference area to provide an indicator of broader secular trends in RTAs. We chose the whole area covered by Glasgow City Council and its surrounding local authorities, partly because the intervention area spanned two local authority areas, and partly to provide a mixture of urban and rural areas and varied designs and densities of road networks for comparison.

### Denominator: road traffic counts

The causes of RTAs are multifactorial, and there is mixed evidence as to whether an increase in road traffic count is associated with an increased number of RTAs,^16^ or whether traffic count data should be included in RTA time-series models.^17^ We extracted traffic count data from three available sources, but the counter locations changed after the opening of the M74 extension from local streets to motorways. However, the available data demonstrated no substantive overall spatio-temporal change in traffic counts in Glasgow. Owing to this potential source of bias, the lack of reliable traffic count for local streets and that traffic count data remained stable in each of the study areas, the time-series models were applied to accident count data only.^18–20^

### Statistical analysis

#### Time-series analysis

An interrupted time-series model was chosen for its ability to assess a series of data points over a continuous time period in order to detect changes in the secular trend at specified time points.^21^ Interrupted time-series regression models with autoregressive integrated moving average (ARIMA) errors were fitted to monthly count data to test the study hypotheses.^22^ ^23^ Log transformations and differencing were applied to achieve time series that were normally distributed and stationary in level and variance. For time series in which some months had zero RTAs, such as those limited to serious and fatal accidents, the series were transformed using an inverse hyperbolic sine function.^24^ Individual models were fitted to each study area and data series. STATA/SE V.14.1 was used for the analysis.

### Model fit

Autocorrelation functions (ACFs) and partial ACFs (PACFs) of each time-series model were used to identify seasonality and guide the initial model building. Detailed residual diagnostics were used to obtain a model with more accurate coefficient estimates. Outliers were identified following visual inspection of the initial models (p<0.05); dummy variables for these outliers were then included and the models rerun.

The Akaike information criterion (AIC) was used as a mechanism to allow us to choose between competing ARIMA error models to broadly assess the fit of the model.^25^ The AIC was also used to assess whether removing outliers from the model improved the overall fit.

### Definition of the intervention effect

#### Motorway construction

Motorway construction began in June 2008 and continued until the opening of the motorway 3 years later. The full construction period was assessed for its impact on RTAs. During the construction period, local roads were closed and diverted, and this immediately and directly affected travel for drivers, pedestrians and cyclists. These abrupt changes to the local road networks may have increased RTAs as local residents travelled unfamiliar routes. This hypothesised impact was modelled as an abrupt and temporary (36 months) intervention effect lasting for the full duration of the construction phase.

#### Motorway opening

The M74 motorway extension opened on 28 June 2011. A ramp intervention effect was used to model any impact of the opening, which was assumed to be gradual and permanent.^26^ Although it could be assumed that the opening of the motorway on a specific date was an abrupt step event with the effect maintained thereafter, this pattern may not be applicable to new transport infrastructure. Changes in human behaviour, daily routines and other adjustments to new infrastructure often take months (or longer) to become fully embedded.^27^ The use of the motorway could therefore be expected to increase gradually following its opening and to be maintained thereafter. Sensitivity analyses were performed to explore the impact of different intervention classifications showing little differences in results.

### Model development

The most appropriate ‘intervention’ classification for interrupted ARIMA time-series models can be guided using the AIC criterion or based on the anticipated impact of an intervention,^28^ as in this case. We also modelled the series using different intervention classifications (step, ramp or ramp-and-step) to reflect alternative ways of theorising the impact of motorway opening as ‘abrupt permanent’, ‘ongoing gradual’ or both ‘gradual’ for a short period (3 months) and then ‘permanent’. These alternative specifications made no material difference to the overall results or goodness of fit of the models.

In addition, we found no change in the variance of the series during the construction period and following the opening, and no change in the seasonal variance of the series when explored using a seasonal decomposition procedure based on Loess (analysed in R V.0.98.1103).^29^

### Casualties

Changes in the number of casualties (pedestrian, driver or rider and passenger) were too small for time-series models. Therefore, changes in the proportion of pooled casualty numbers by road user and study area are reported for serious and fatal casualties. Changes in proportions of casualty number by study areas were measured using analysis of variance (ANOVA) and, if significant, pairwise comparisons were contrasted between individual study areas.

## Results

### General trends in the number and severity of accidents

**Figure 2:**
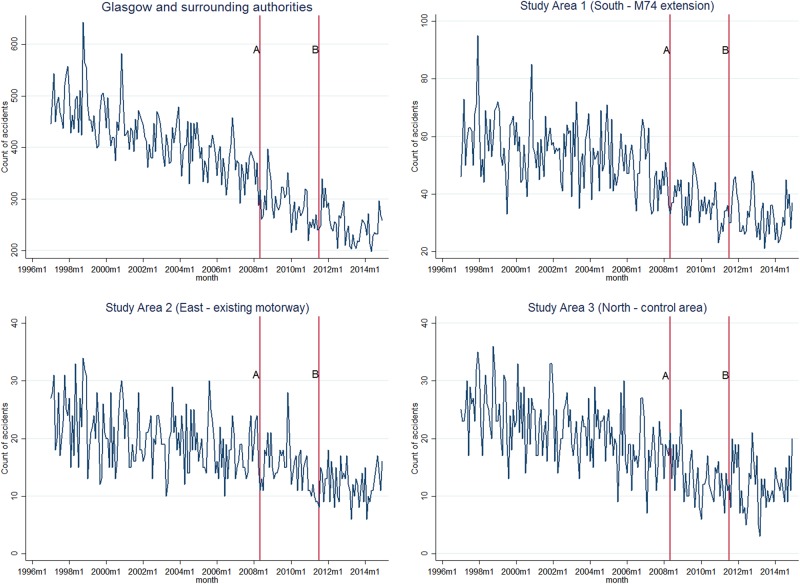
Monthly trends in RTAs (all accidents) by study area, 1997–2014. RTAs, road traffic accidents.

All study areas experienced a reduction in the annual number of RTAs for all accidents during the period 1997–2014 (figure 2): Glasgow and surrounding authorities experienced a 50.6% reduction (from 5901 to 2914), with reductions of 50.7% in the South (from 758 to 374), 49.3% in the East (from 292 to 148) and 50.5% in the North (from 315 to 156). When analysis was limited to serious or fatal accidents, there were greater proportional decreases across all areas (see online supplementary table S1).

10.1136/jech-2016-207378.supp1 Supplementary table Annual number of RTAs by severity and study area, 1997 to 2014.

There was large variation in the total monthly accident count within each study area, but an overall downward trend across all areas. The decline was more apparent in the South (intervention) area, than in the East and North study areas.

### Impact of the M74 motorway extension on the number of accidents

#### All accidents

Table 1 shows the results of the time-series models. The column ‘estimate’ provides the monthly change in the number of RTAs; a negative number indicating a month-by-month reduction in the number of RTAs for that area and size of that reduction.

**Table 1:** Interrupted time-series regression with ARIMA errors for (a) all accidents and (b) serious and fatal accidents only, by study area

		Parameter	Estimate (95% CI)	p Value	Parameter	Estimate (95% CI)	p Value
Study area	Series	(a) All accidents			(b) Serious and fatal accidents only		
Glasgow and surrounding local authorities	Change in series for full study period	Log ar(7 9 10 12) sarima(0,1,0,12)	−0.042	<0.0001	Log ar(1 7 12) sarima(0,1,0,12)	−0.074	<0.0001
			(−0.054 to −0.030)			(−0.107 to −0.041)	
	Change in series during M74 construction period		−0.066	<0.0001		0.046	0.337
			(−0.098 to −0.035)			(−0.048 to 0.139)	
	Change in series following opening of M74 motorway extension		−0.001	0.295		0.0027	0.385
			(−0.003 to 0.001)			(−0.003 to 0.009)	
Intervention area (South, M74 extension)	Change in series for full study period	Log ar(1 12 13) sarima(0,1,0,12)	−0.41	0.003	Log ma(1 12) sarima(0,1,0,12)	−0.088	<0.0001
			(−0.069 to −0.014)			(−0.125 to −0.063)	
	Change in series during M74 construction period		−0.026	0.558		−0.082	0.705
			(−0.116 to 0.063)			(−0.451 to 0.299)	
	Change in series following opening of M74 motorway extension		−0.001	0.951		−0.003	0.67
			(−0.006 to 0.006)			(−0.017 to 0.011)	
Comparator area (East, M8 motorway)	Change in series for full study period	Log ar(1 12) sarima(0,1,0,12)	−0.053	0.001	Log ma(12) sarima(0,1,0,12)	−0.119	<0.0001
			(−0.083 to −0.022)			(−0.163 to −0.075)	
	Change in series during M74 construction period		0.065	0.264		−0.044	0.831
			(−0.049 to 0.179)			(−0.446 to 0.358)	
	Change in series following opening of M74 motorway extension		0.005	0.142		0.012	0.255
			(−0.002 to 0.013)			(−0.008 to 0.032)	
Control area (North)	Change in series for full study period	Log ar(11) sarima(0,1,1,12)	−0.054	0.002	Log ma(12) sarima(0,1,0,12)	−0.074	<0.0001
			(−0.088 to −0.019)			(−0.115 to −0.033)	
	Change in series during M74 construction period		−0.353	0.029		−0.08	0.722
			(−0.669 to −0.037)			(−0.521 to 0.361)	
	Change in series following opening of M74 motorway extension		−0.029	<0.0001		0.0001	0.994
			(−0.043 to −0.016)			(−0.024 to 0.024)	

ARIMA, autoregressive integrated moving average.

Interrupted time-series regression found a significant decrease in the total number of RTAs during the period 1997–2014, both in Glasgow and surrounding authorities as a whole (−0.042, 95% CI −0.054 to −0.030) and in the South (−0.41, 95% CI −0.069 to −0.014), East (−0.05, 95% CI −0.083 to −0.022) and North study areas (−0.054, 95% CI −0.088 to −0.019; table 1a). Evidence of a further decrease in the temporal trajectory of RTAs following motorway opening was shown in the North control area (−0.029, 95% CI −0.043 to −0.016), but not in Glasgow and surrounding authorities or in the South or East study areas.

#### Serious and fatal accidents

Table 1b shows that each area had a significant decrease in the number of serious and fatal RTAs during the full study period 1997–2014. However, the time-series analysis showed no significant reduction in serious and fatal RTAs associated with either the construction or the opening of the M74 motorway extension (table 1b).

### Trends in RTA casualties by road user and study area

Table 2 presents the pooled number of serious and fatal casualties resulting from an RTA by type of road user and study area, and the percentage change in these, for the 3-year periods 1997–1999 and 2012–2014. It shows that each area experienced substantial decreases in the numbers of casualties in each category (pedestrian, driver or rider and passenger). The reduction in number of pedestrian casualties in the South was tempered relative to Glasgow and surrounding authorities (T=3.25, 95% CI 0.016 to 0.158). There were no differences in proportions between study area for driver or rider and passengers. Numbers of accidents involving cyclists only were too few for analysis.

**Table 2:** Change in the number and proportion of serious and fatal RTA casualties by road user and study area from 1997–1999 to 2012–2014

	Pedestrian			Driver or rider			Passenger		
Study area	1997–1999	2012–2014	% Change	1997–1999	2012–2014	% Change	1997–1999	2012–2014	% Change
Glasgow and surrounding local authorities	1703	571	−66.5	1504	585	−61.1	863	208	−75.9
Intervention area (South, M74 extension)	210	101	−51.9	121	61	−49.6	79	17	−78.5
Comparator area (East, M8 motorway)	111	39	−64.9	54	28	−48.1	32	11	−65.6
Control area (North)	122	38	−68.9	59	19	−67.8	33	5	−84.8
	ANOVA p=0.013 F=3.60*			ANOVA p=0.157 F=1.74			ANOVA p=0.535 F=0.73		

*Differences between each study area were tested (six comparisons) and remained significant only for differences between Glasgow and surrounding local authorities and intervention area (South, M74 extension) (T=3.25, p=0.007, 95% CI 0.016 to 0.158).

ANOVA, analysis of variance.

## Discussion

### Main findings

This study evaluated the impact of new urban transport infrastructure on the number of RTAs in the local area. We found no clear evidence that the M74 motorway extension altered the already declining incidence of RTAs overall, either during construction or following its opening. We also found no evidence of an effect on serious and fatal accidents in particular. We therefore found no evidence to support either the claims or the counterclaims made prior to construction about the potential increases or decreases in accidents on local streets.^11^

The North study area showed a significant decrease in the temporal trajectory of RTAs following the opening of the M74 motorway. There are two reasons why it is highly unlikely that this was due to the M74 motorway itself. First, no such change was observed in the region as a whole, in the South study area surrounding the M74 motorway extension, or in the East study area containing an existing motorway. If such an observed association were causal, it would most likely have been observed first and most strongly in the area surrounding the new motorway, and second on other major roads in the city that would have experienced a significant change in traffic flow; neither of these effects was apparent. Second, the limited traffic count data available suggested that the M74 extension had little impact on trends in the number of vehicles travelling in the city as a whole.

It is important to consider other city-wide programmes which may have influenced RTAs. For example, the North area contained a local transformational regeneration zone which from 2010 implemented new housing, community facilities and improved green space.^30^ It also benefited from the reopening of a disused railway line, improvements to two cycling and walking paths to the city centre, and the introduction of bus lanes. These may have contributed to the additional decrease in RTAs observed in the North.

### Comparison with existing literature

Over the course of the study period, RTAs declined significantly across all study areas. This corroborates the findings of other UK analyses.^1^ ^7^ We did not find any recent studies that have evaluated the impact of new urban motorways of this kind on the incidence of RTAs. A data summary of evidence showed before and after reduction in RTAs following new motorway construction across Western Europe and North America between 7% and 9%.^10^ A further North American study evaluating improvements to existing motorway infrastructure (in the form of increasing and widening lanes) showed no impact on the number of RTAs.^31^ Reviews of a variety of interventions suggest that red light cameras,^32^ 20 mph zones,^33^ average speed enforcement^34^ and other types of speed camera,^35^ graduated driver licensing^36^ and increasing the visibility of pedestrians and cyclists^36^ are effective in reducing RTAs. However, none of these was strategically implemented in the area adjacent to the M74 motorway extension, highlighting the possibility of a missed opportunity to augment the motorway construction project with additional interventions that could have further reduced RTAs in the local area.

RTAs result from a large number of casual factors, and our study highlights the weakness of natural experiments where there are many other contributing factors which may be influential. We have described factors in different areas, such as recent investment in housing, cycle networks and bus lanes in the North area. There are a large number of other factors that contribute to RTAs, such as alcohol^37^ and changes to street lighting.^13^

The intervention area saw an attenuated reduction in the number of pedestrian casualties relative to Glasgow and surrounding authorities for the periods 1997–1999 and 2012–2014, not time period specific to the M74 motorway extension opening.

### Strengths and limitations

We extracted RTA data from the STATS19 portal; although these data are widely used in research,^7^ ^13^ ^28^ they have limitations. They do not record all RTAs and rely upon the accuracy of police officers in correctly providing the exact location of the accident.^13^ Importantly, in the UK, there is no legal obligation to report a traffic collision to the police, and this may lead to an under-reporting of RTAs,^1^ particularly those resulting in only minor injuries.^38^ However, it is unlikely that there were any systematic differences in, or changes in, the accuracy of reporting of accidents between study areas during the study period, particularly since a single police force covers the entire area.

We compared data from three areas within Glasgow, adjacent to new or existing transport infrastructure, to assess the extent to which any change in the incidence of RTAs might be attributable to the opening of the M74 motorway extension. This did entail certain assumptions in the way the impacts of motorway construction and opening were modelled, but using alternative plausible classifications of intervention effect did not change the overall results. The number of casualties was greater at baseline and postintervention in the South area compared with the East and North areas; the analysis we conducted explored changes in the number of accidents at a landscape level using ANOVA. However, this does highlight a limitation of this kind of natural experiment where it is difficult to define an identical comparator area in terms of road infrastructure, size, sociodemographic characteristics and number of casualties.

We made considerable efforts to obtain accurate and reliable traffic count denominator data for the time-series models. Regardless, without traffic count data included in our models, the time-series analysis showed no significant reduction in accidents that could clearly be attributed to the M74 motorway extension. Best available data, published by Transport Scotland in their ‘16 Week After Opening Review’, described increases and decreases of traffic flow on local roads; increases on the main arteries to the motorway and decreases on some local streets. The report stated the M74 motorway extension had satisfied the original objectives of the scheme in terms of its anticipated traffic flow and transferring vehicles from other roads and motorways in the region.^39^ Importantly, the ARIMA procedure used in our time-series analysis provides a rigorous method for assessing the impact of interventions such as new transport infrastructure on outcomes measured using count data.

## Conclusions

Our results suggest that policymakers cannot necessarily appeal to a likely reduction in RTAs in justifying the construction of new urban motorway infrastructure. It may be taken for granted that new road infrastructure alone will reduce RTAs, as was argued before the construction of the M74 extension, but we found no evidence that the key strategic and economic development objective to improve road safety and reduce road accidents on local streets had been achieved by 2014. The intervention may have had a number of other important impacts, for example, on air pollution and active travel, but these were not modelled in this paper and are worthy of further investigation in their own right. In addition, accident rates are subject to changes at local level, and although the motorway extension may not have reduced the overall number of RTAs, it remains possible that it may have contributed to changing their spatio-temporal distribution—for example, by shifting the burden of accidents into or out of poorer neighbourhoods. This will be examined in future research. Global urbanisation is proceeding at pace; however, evidence of infrastructure changes is lacking. Our study provides novel and important findings for future developments.

**What is already known on this subject**

- Building new urban motorways is controversial, with arguments (including public health arguments) both for and against construction.
- There is little evidence of the impact of new urban motorway infrastructure on road traffic accidents.
- The 5-mile M74 motorway extension opened in June 2011 in Glasgow, Scotland, and provided a natural experimental opportunity to evaluate its impact on temporal trends in road traffic accidents.

**What this study adds**

- We found no evidence that the motorway extension had altered the already decreasing trajectory in the incidence of road traffic accidents, either during construction or following its opening.
- Our findings suggest that in planning future investment, it should not be taken for granted that new road infrastructure alone will reduce RTAs in local areas.
