# Supplementary material for: Effects of new urban motorway infrastructure on road traffic accidents in the local area: a retrospective longitudinal study in Scotland
Source: J Epidemiol Community Health. 2016 Jun 8;70(11):1088–95. doi: 10.1136/jech-2016-207378 (PMC5541177; doi:10.1136/jech-2016-207378)
Supplement: Supplementary table — Annual number of RTAs by severity and study area, 1997 to 2014. [file jech-2016-207378supp_table.pdf]

Supplementary Table 1: Annual number of RTAs by severity and study area, 1997 to 2014.

	Glasgow & surrounding local authorities		Study Area 1 – South Intervention Area		Study Area 2 – East Comparator Area		Study Area 3 – North Control Area	
	All	Serious & Fatal	All	Serious & Fatal	All	Serious & Fatal	All	Serious & Fatal
1997	5,901	1,223	758	129	292	70	315	70
1998	5,956	1,229	708	123	300	68	312	73
1999	5,469	1,174	685	131	241	49	268	63
2000	5,429	1,030	680	84	258	52	285	46
2001	5,198	947	665	111	233	44	274	52
2002	5,024	912	665	103	243	43	266	47
2003	4,951	861	660	97	233	41	242	48
2004	4,865	707	650	80	234	31	256	34
2005	4,613	629	614	68	232	32	239	43
2006	4,504	725	615	107	209	32	226	30
2007	4,279	621	535	68	200	23	198	31
2008	3,885	713	496	91	208	34	208	46
2009	3,620	584	459	61	203	31	149	20
2010	3,283	515	424	63	175	26	147	28
2011	3,234	463	417	57	133	15	155	28
2012	3,061	467	400	60	155	31	141	23
2013	2,733	368	362	48	135	23	119	15
2014	2,914	427	374	55	148	20	156	22
